# How Time, Narrative, and Discomfort Shape Learning: A Realist Evaluation of Behavioral Science Education for Medical Students

**DOI:** 10.5334/pme.2332

**Published:** 2026-05-05

**Authors:** Junji Haruta, Toshiaki Kikuchi, Junko Kitanaka, Makoto Kurata

**Affiliations:** 1 Medical Education Center, Keio University School of Medicine, Tokyo, Japan; 2 Center for General Medicine Education, Keio University School of Medicine, Tokyo, Japan; 3 Department of Neuropsychiatry, Keio University School of Medicine, Tokyo, Japan; 4 Department of Human Sciences, Keio University, Tokyo, Japan; 5 Faculty of Medicine, Tokyo Medical School, Tokyo, Japan

## Abstract

**Introduction:**

Medical students are increasingly expected to navigate uncertainty, emotional complexity, and value conflicts, yet little is known about how behavioral science education fostered these capacities. This study aimed to identify the mechanisms through which a third-year behavioral science course influenced students’ cognitive, emotional, and relational development.

**Methods:**

We conducted a realist evaluation of a course that integrated structured (Cognitive Behavioral Therapy; CBT)-based reflection, narrative immersion using complex clinical cases, and conceptual framing from the humanities and social sciences. Slow Education served as a sensitizing concept in developing initial program theories. Data were drawn from written reflections, group discussions, classroom observations, and field notes from two cohorts of students (N = 210). Abductive and retroductive coding strategies were used to refine context–mechanism–outcome (CMO) configurations.

**Results:**

We identified five learning mechanisms: (1) emotional articulation that enabled readiness for deeper engagement; (2) narrative destabilization of biomedical certainty; (3) positional reframing supported by structured questioning; (4) epistemic disruption that fostered tolerance for ambiguity; and (5) conceptual reframing that legitimized discomfort and supported meaning-making. These mechanisms operated sequentially, forming a cumulative learning pathway. A certainty-seeking response functioned as a competing mechanism for some students, leaving intended mechanisms unactivated under particular contextual conditions.

**Discussion:**

The findings demonstrated how behavioral science education activated interconnected mechanisms that supported students’ development of reflective capacity, perspective-taking, and tolerance for ambiguity. The resulting middle-range explanation clarifies why structured emotional work, narrative complexity, guided questioning, and conceptual scaffolding enabled deeper engagement with the psychosocial dimensions of clinical practice.

## Introduction

Medical students routinely encounter uncertainty, emotional tension, and conflicting values during clinical training [1,2]. Yet traditional biomedical curricula offer limited opportunities to engage with these forms of complexity, often prioritizing accurate diagnosis and efficient management over reflection, dialogue, and relational understanding. As a result, learners may enter clinical environments insufficiently prepared for the emotional and ethical dimensions of patient care—areas that require perspective-taking, critical self-reflection, and tolerance for ambiguity [3,4].

In response to these challenges, medical education has increasingly incorporated insights from the humanities and social sciences, including anthropology, psychology, and sociology [5,6,7]. These approaches aim to help learners make sense of illness experiences, social determinants, and cultural framings of health [8,9]. Recent scholarship has provided conceptual models for integrating such perspectives into curricula. For example, Gülpınar and Tanrıöver describe three domains essential for behavioral and social sciences integration: (1) contextualized and reflective practice, (2) awareness of health-system and structural forces, and (3) ecological understandings of health and illness [10]. Empirical work further suggests that humanities-informed pedagogies can support humanistic attitudes, relational skills, and ethical sensitivity among medical trainees [11,12].

Additionally, a significant gap in medical education is the pervasive ‘certainty-seeking’ tendency among students, which often leads to the avoidance of ambiguity and discomfort. While traditional curricula prioritize rapid knowledge acquisition (efficiency), they often fail to prepare students for the inherent uncertainty of clinical practice. To address this, we designed an intervention based on transformative learning and experiential learning cycles. By intentionally inducing cognitive disequilibrium—a state where learners encounter ‘troublesome knowledge’ that cannot be explained by their existing biomedical schemas—we motivate students to engage with behavioral science not as abstract theory, but as a necessary tool for navigating professional discomfort

Despite the development of such pedagogical approaches, our understanding of how behavioral science education fosters meaningful learning remains limited. Prior studies have rarely examined the specific mechanisms through which pedagogical strategies activate cognitive, emotional, or relational shifts in learners, nor have they clarified the contextual conditions that enable or constrain such shifts. This lack of mechanism-focused inquiry poses a barrier to the design and transferability of behavioral science curricula across institutions and cultures.

To address this gap, we conducted a realist evaluation of a third-year behavioral science course that combined three pedagogical elements: (1) structured cognitive-behavioral reflection, (2) immersion in complex narrative clinical cases, and (3) conceptual framing using theories from the humanities and social sciences. Although the course design was informed by the educational philosophy of “Slow Education,” we deliberately treated it not as a prescriptive theory but as a sensitizing concept that draws attention to the pedagogical value of time, intentional pausing, dialogic engagement, and dwelling with uncertainty [13]. These ideas guided the development of our initial program theories (iPT) regarding how the course might support learners’ readiness for the emotional and epistemic challenges of patient care.

## Theoretical framework

This study drew on realist evaluation to explore how a behavioral science course supports medical students’ learning. Realist evaluation is grounded in the premise that educational outcomes arise from interactions between contexts and mechanisms, rather than from interventions alone [14]. The framework’s central question—“*What works, for whom, in what circumstances, and why?*”—is well suited to behavioral science education, where learning depends on learners’ reasoning, emotional engagement, and interpretation of complex social situations.

To guide the development of our iPTs, we used Slow Education as a *sensitizing concept* rather than a formal theoretical framework [13]. Slow Education emphasizes the pedagogical value of time, intentional pausing, dialogue, and dealing with uncertainty. These ideas informed our hypotheses about how structured reflection, narrative immersion, and conceptual framing might activate particular learning mechanisms. For example, we anticipated that slowing down learners’ engagement with emotionally charged or ambiguous cases would allow them to articulate cognitive–emotional responses, question taken-for-granted assumptions, and engage more deeply with others’ perspectives.

In addition, theories related to reflective practice, narrative learning, and cognitive–emotional processes sensitized our attention to potential mechanisms [15,16,17,18,19]. CBT (Cognitive Behavioral Therapy)-based reflection highlights the role of identifying and reframing emotional and cognitive patterns; narrative-based approaches suggest that complex stories can destabilize overly biomedical interpretations; and sociocultural theories provide a lens for understanding how conceptual tools help learners make meaning of discomfort or uncertainty. These theoretical perspectives together informed the mechanism-focused hypotheses that guided our realist analysis.

## Research Questions

Guided by realist methodology, this study addressed the following questions:

1. What mechanisms are triggered when medical students participate in structured CBT-based reflection, narrative case immersion, and conceptual framing activities within a behavioral science course?
2. Under what contextual conditions do these mechanisms lead—or fail to lead—to changes in emotional literacy, perspective-taking, and tolerance for ambiguity?
3. How do empirical data from students’ reflections and discussions refine the iPTs derived from the sensitizing concept of Slow Education?

## Methods

### Study design

We conducted a realist evaluation to examine how a third-year behavioral science course supported medical students’ learning. Realist evaluation assumes that educational outcomes arise from the interaction between contexts (C), mechanisms (M), and outcomes (O), and seeks to explain *what works, for whom, in what circumstances, and why* [20]. This approach is particularly suited to pedagogical interventions that activate cognitive and emotional processes rather than deliver standardized knowledge.

Our analysis was guided by the RAMESES II reporting standards for realist evaluations and proceeded iteratively through developing iPTs, testing them against empirical data, and refining them into final CMO configurations [21].

### Educational context

The study took place at a private medical school in Japan where behavioral science is vertically integrated throughout the curriculum. The focal course (Behavioral Science II) is delivered in the third-year—just prior to clinical clerkships—when students typically begin grappling with professional identity formation, increasing workload, and heightened emotional and academic demands. Traditional biomedical teaching at this stage offers limited opportunities to reflect on patients’ lived experiences, sociocultural contexts, or emotional complexity, creating a pedagogical rationale for a behavioral science intervention.

### Description of the intervention

The course comprised three interrelated components:

1. **Structured CBT-based reflection (half-day workshop):**
  Students completed cognitive–emotional worksheets guided by a psychiatrist, identifying recent emotionally salient experiences and analyzing the thoughts, feelings, and behaviors associated with them.
2. **Narrative case immersion (three case discussions over 1.5 days):**
  Cases were co-developed by a general practitioner and medical anthropologists and included ambiguity, moral tension, family dynamics, and sociocultural factors. Small-group and whole-class discussions encouraged exploration of multiple perspectives.
3. **Conceptual framing (short lectures):**
  Key concepts from the humanities and social sciences—such as illness narratives, structural competency, cultural framing, and negative capability—were introduced immediately after discussions to support sense-making.

To guide theory development, we treated Slow Education not as a formal framework but as a sensitizing concept emphasizing intentional pausing, dialogic deliberation, and dwelling with uncertainty. These features informed our initial assumptions about how learning might occur.

### Participants

Participants were all third-year medical students enrolled in the mandatory course in 2023 (n = 114) and 2024 (n = 110). Of these, 102 and 98 students respectively submitted complete reflective assignments and contributed data for analysis. Students were informed about the study, and written consent was obtained. Participation had no bearing on course grades.

Gender distribution was 33%–38% female, consistent across cohorts. No additional demographic data were collected, in line with institutional norms, though we acknowledge that this limits insight into potential subgroup differences.

### Researcher positionality

The research team consisted of:

- A general practitioner (first author) with experience in community-based practice and medical anthropology
- Two medical anthropologists experienced in qualitative and narrative analysis
- A psychiatrist with expertise in CBT and reflective practice

This interdisciplinary composition shaped our sensitivity to different types of mechanisms (cognitive, emotional, relational, sociocultural). Because the first author co-facilitated the course, reflexive memos were kept throughout data collection and analysis to mitigate interpretive bias.

### Data sources

We collected multiple qualitative data sources to enable triangulation:

- Pre-class assignments (written responses to case prompts and preparatory questions)
- Small-group discussion notes (student-generated outputs)
- Classroom observations (30–40 hours of field notes)
- Immediate post-session reflections
- Final reflective essays (primary dataset)
- Faculty field notes (anthropologists and GP co-facilitators)

Across both cohorts, the dataset comprised approximately 220 reflective essays and >200 pages of notes.

### Development of initial program theories (iPTs)

iPTs were constructed prior to data analysis and informed by (a) sensitizing concepts from slow education (intentional pausing, narrative dwelling, and tolerance for uncertainty), (b) literature on CBT-based emotional articulation, (c) theories of narrative learning and sociocultural framing, and (d) instructors’ experiential knowledge. These iPTs articulated hypothesized relationships between contexts, mechanisms, and outcomes and served as propositions to be tested and refined through realist analysis (Table 1).

**Table 1:** Initial Program Theories (iPTs).


iPT NO.	CONTEXT ASSUMPTION	ANTICIPATED MECHANISM (INTERNAL PROCESS)	EXPECTED OUTCOME

iPT1	Students often lack structured emotional reflection	CBT-guided emotional articulation will prepare students for deeper narrative engagement	Increased readiness for discussion and self-awareness

iPT2	Students hold biomedical certainty	Narrative immersion will destabilize fixed biomedical assumptions	Greater narrative sensitivity

iPT3	Students default to self-centric interpretive habits	Socratic questioning will facilitate positional reframing	Enhanced perspective-taking

iPT4	Students tend to avoid uncertainty	Encounters with unresolved or ambiguous cases will disrupt epistemic certainty	Improved tolerance for ambiguity

iPT5	Students lack conceptual tools to make sense of discomfort	Conceptual framing will legitimize discomfort and support meaning-making	Strengthened reflective capacity


### Data analysis

Data analysis followed the iterative, abductive–retroductive process recommended for realist evaluation:

#### 1. Preliminary coding (abductive phase)

The first author conducted the initial open code on the dataset. Codes were both theory-informed (e.g., “emotional reframing,” “perspective shift”) and inductively derived (e.g., “discomfort with ambiguity,” “questioning correctness”). Through discussion among all authors, we refined a shared codebook.

#### 2. CMO configuration (retroductive phase)

We identified candidate mechanisms as students’ internal reasoning or emotional processes, not as instructional activities. Mechanisms were inferred when:

- students described changes in assumptions, interpretations, or emotional responses
- shifts occurred within identifiable contextual conditions
- multiple data sources converged (triangulation)

Each candidate mechanism was mapped to C (context) and O (outcome) components, generating provisional CMO configurations.

#### 3. Refinement of program theories

We compared iPTs with observed CMOs to determine where expectations were confirmed, contradicted, or required modification. Through iterative comparison across cohorts, we refined a set of five recurring CMO configurations that demonstrated explanatory power.

#### 4. Triangulation and credibility checks

- Student essays were compared with observation notes to validate inferred mechanisms
- Negative or disconfirming cases were examined to identify competing or unactivated mechanisms
- The full analytic process was discussed among all four authors to enhance trustworthiness

To support transparency in how the CMO configurations were developed and refined, we compiled representative excerpts from student reflections for each mechanism. These illustrative data are provided in Additional file 1.

### Ethical considerations

The study was approved by the Research Ethics Committee of Keio University (Approval No. 2025-1027). Participation was voluntary, and data were anonymized. We adhered to institutional guidelines for the conduct and reporting of educational research.

## Results

Our analysis identified five core learning mechanisms that explained how the course supported students’ cognitive, emotional, and relational development. The analysis also revealed a competing, unactivated mechanism -certainty-seeking- that inhibited learning for some students. The mechanisms refined and, in some cases, extended our iPTs. Each mechanism is presented in terms of its context (C), mechanism (M)—defined as students’ internal reasoning or emotional processes—and outcome (O) (Table 2). Additional file 1 contains extended examples from both cohorts to allow readers to examine the qualitative evidence in greater depth.

**Table 2:** Final CMO Configurations Identified through Realist Analysis (Post-course Results).


				REPRESENTATIVE DATA (EXCERPTED)

**Descriptive titles for each mechanism**.	**Key Contexts (C)**	**Refined Mechanism (M)**	**Observed Outcomes (O)**	*Student A (2023): I learned that the cognitive behavioral model is built on the idea that cognition affects mood and behavior. Writing about a time I felt frustrated during club activities helped me see how my assumptions shaped my reactions*. *Student B (2024): By identifying the thoughts behind my irritation toward a peer, I realized I could reframe the situation and respond more constructively to patients. As a future physician, I realized the importance of being mindful of my own mental health. Using CBT helped me reflect and manage negative thought patterns*.

Cognitive Decentering	Emotional burden; lack of previous reflection	The process of “naming” one’s emotions as objective data, allowing students to gain metacognitive distance and prepare for self-reflection.	Increased openness; self-awareness	*Student C (2023): Reading the case of a lawyer with Guillain-Barré syndrome who later faced stomach cancer made me realize how differently patients may perceive treatments. Through group discussion, I recognized the importance of understanding patient backgrounds and perspectives*. *Student D (2024): I learned the value of imagining patients’ lived realities. Simply seeing “non-adherence” to medication might evoke frustration, but imagining the reasons behind such behaviors fosters empathy and better care*.

Schema Dissonance	Biomedical-centric training	Encountering complex narratives that disrupt the “diagnosis-as-solution” frame, forcing an epistemic shift through immersion in lived experiences.	Recognition of patient lived realities	*Student E (2023): What I learned most in behavioral science was how, by studying in a closed environment like medical school and later working in a highly specialized healthcare setting, I may unconsciously lose the “patient’s perspective.” To remain a doctor who can see patients in a balanced way, I realized the importance of having regular opportunities—like this course—to deeply consider patients’ backgrounds. I spend much of my time in the lab doing research, immersed in highly specialized discussions even more than typical clinical settings, which makes me even more prone to losing sight of general perspectives. If we are to advance medicine and save more patients, we must intentionally make time to broaden our view*. *Student F (2024): I learned that when I’m truly stuck as a physician, it’s essential to involve more people in the problem-solving process. Also, neither patients nor their caregiving families should be left isolated. I came to understand that even the best treatments are less effective when someone is socially isolated*. *Student G (2024): We are repeatedly taught from elementary school that it’s important to view things from various perspectives, and I’ve always believed that. However, through this behavioral science course, I realized I wasn’t truly seeing things from multiple angles. I tended to interpret things through the lens of my own values and beliefs, and I noticed that I had unconsciously excluded perspectives I couldn’t understand or imagine*.

Cognitive De-shackling/Perspective-taking	Self-centric interpretive habits	A conscious realization of one’s own unconscious biases (physician-centeredness) through structured inquiry, enabling the simulation of the “other’s” logic.	Perspective-shifting; empathy	*Student H (2023): In other courses, we primarily focus on diagnostics and treatment protocols, so I’ve come to believe that the most important thing is to provide evidence-based, scientifically appropriate care. But in behavioral science, I came to recognize how critical the emotional bond between doctor and patient is to successful treatment outcomes. I want to explore how best to build trust in daily relationships. I was particularly struck by the realization that patients may hold values completely different from any I’ve encountered. As a physician, even when facing value differences with patients, we must rationally understand their values and decide on a treatment plan, but I can easily imagine myself reacting emotionally. I want to keep in mind that the values I know are only a small part of the spectrum*. *Student I (2024): I felt how difficult it is to provide truly patient-centered care, especially in end-of-life settings. Balancing a patient’s wishes against what is medically right is a tough issue. Respecting a patient’s autonomy and how they want to live their remaining life can offer peace and dignity. But if the patient’s choices aren’t medically appropriate, it could lead to deterioration and more suffering. If their decisions also burden their family, relationships might suffer. On the other hand, focusing on medically “correct” care helps improve the physical condition and makes treatment easier from the physician’s standpoint. However, that could lead to ignoring the patient’s wishes, eroding trust. To resolve this, I think flexibility is necessary—listening closely to the patient’s reasoning and negotiating the best path forward. In the case we studied, the physician didn’t force treatment but respected the patient’s stance, which helped build trust and led to satisfactory care. I realized that in end-of-life care, what’s medically right isn’t always the top priority for the patient*.

Tolerance of Ambiguity	Expectation of clear answers	The suppression of the impulse for premature closure, allowing students to remain in a state of “not knowing” while maintaining attentive observation.	Tolerance for ambiguity	Student J (2023): I felt I understood the meaning of the term “troublesome knowledge” mentioned by our instructor. In the case presented, the focus was not on the disease itself but on the patient’s family dynamics and social background. It would be ideal to understand those aspects easily, but in real home visits, it takes time and persistent dialogue to earn trust and grasp such complexity. I recalled a passage from the book “Being Here is Painful,” which said that what’s most important in connecting hearts isn’t diagnosing or treating a disease but simply being present and becoming part of that space. To truly understand what matters, it’s not about efficiency—it requires time and patience. Student L (2024): The behavioral science assignments didn’t have clear answers, so I had to think deeply from various angles, which was often challenging. I learned about the term “negative capability” for the first time and felt it would be a necessary trait for me as a future physician. We had many opportunities for discussion, and I realized how diverse perspectives can be, even among classmates. I want to become someone who, when faced with unsolvable issues, values dialogue with fellow doctors, interprofessional colleagues, patients, and families, and can consider problems from many dimensions

**Conceptual Reframing**	Feeling “inadequate” during discomfort	Utilizing concepts like *Negative Capability* to legitimize discomfort not as a failure, but as an essential element of professional identity formation.	Meaning-making; emotional legitimation	


### Mechanism 1: Emotional articulation enabling readiness for deeper engagement

*(Refined from iPT1; emotional literacy emerged as a prerequisite for subsequent mechanisms.)*

#### Context

At the beginning of the course, many students were juggling increasing academic demands, extracurricular leadership responsibilities, and uncertainty about their future roles as clinicians. Despite this emotional burden, few had prior structured opportunities to reflect on their internal states.

#### Mechanism (internal process)

The CBT-based reflective exercise prompted students to identify and name their emotional responses, recognize cognitive patterns, and take a metacognitive stance toward their reactions. Students described gaining distance from their emotions and becoming more aware of how their assumptions shaped their interpretations of clinical situations.

#### Outcome

This emotional articulation generated a sense of readiness for deeper engagement in the subsequent narrative discussions. Students reported entering group dialogue with heightened openness and capacity to consider perspectives other than their own.

*“Understanding how my thoughts shaped my frustration helped me see how patients’ emotions might also have understandable reasons.” (Student 2024)*

### Mechanism 2: Narrative destabilization of biomedical certainty

*(Aligned with iPT2 but revealed a stronger destabilizing effect than anticipated.)*

#### Context

Students had been primarily socialized within a biomedical framework that emphasized correctness, evidence-based reasoning, and efficient problem-solving. Their prior learning afforded limited exposure to contextualized, value-rich patient narratives.

#### Mechanism (internal process)

Engagement with rich, ambiguous clinical narratives—featuring family dynamics, moral tension, and culturally situated decision-making—challenged students’ default reliance on biomedical interpretations. Students began to question the assumption that medically “correct” decisions were always aligned with patient priorities.

#### Outcome

Students developed greater narrative sensitivity, showing increased recognition that patients’ decisions are shaped by lived realities and sociocultural contexts, not simply biomedical considerations.

*“When I imagined her daily life rather than just the disease, the ‘right’ choice looked very different.” (Student 2023)*

### Mechanism 3: Guided questioning prompting positional reframing

*(Supported iPT3 and clarified the importance of structured questioning techniques.)*

#### Context

Although students were familiar with the idea of adopting “the patient’s perspective,” they often defaulted to self-centric interpretations and struggled to imaginatively inhabit others’ viewpoints without scaffolding.

#### Mechanism (internal process)

Facilitators used four structured types of questions—open, positional, introspective, and counterfactual. These questions helped students interrogate their assumptions, recognize their own positionality, and imaginatively shift between perspectives. The questioning destabilized initial judgments and created space for reinterpretation.

#### Outcome

Students described deliberate perspective shifting, moving from “What should this physician do?” to “Why might this patient act this way?” This reframing expanded their cognitive flexibility and capacity for empathy.

*“I thought I knew how to see from another person’s viewpoint, but I realized I had only been seeing through my own values.” (Student 2024)*

### Mechanism 4: Epistemic disruption fostering tolerance for ambiguity

*(iPT4 was largely confirmed; however, competing mechanisms were identified.)*

#### Context

Medical students were accustomed to learning environments where clear answers existed and uncertainty was minimized. They expressed discomfort when confronted with situations lacking definitive solutions.

#### Mechanism (internal process)

Cases intentionally designed with unresolved elements and conflicting values triggered epistemic disruption—a recognition of the limits of medical knowledge and of the presence of competing “right” answers. Students described becoming aware of their impulse to seek closure and learning to withhold premature judgment.

#### Outcome

Most students developed increased tolerance for ambiguity, acknowledging that uncertainty is inherent to clinical practice and does not necessarily indicate personal inadequacy.

*“Not having a clear answer was uncomfortable, but it made me think more deeply about what mattered most to the patient.” (Student 2024)*

#### Competing/unactivated mechanisms

A minority of students demonstrated avoidance responses, such as retreating to biomedical correctness or disengaging from discussion. These cases illustrated a competing mechanism: certainty-seeking, which inhibited learning.

### Mechanism 5: Conceptual reframing legitimizing discomfort and supporting meaning-making

*(Refined iPT5; theories acted not only as cognitive tools but also as emotional validation.)*

#### Context

Students initially interpreted their confusion, discomfort, or emotional tension as signs of inadequacy or lack of knowledge.

#### Mechanism (internal process)

Concepts such as negative capability, structural competency, and troublesome knowledge provided language and theoretical framing for students to understand and legitimize their experiences of discomfort. Rather than dismissing their confusion, students learned to reframe it as a productive and expected part of professional development.

#### Outcome

Students engaged in meaning-making, reinterpreting their uncertainty as a space for reflection rather than as failure. They connected personal emotional responses with broader sociocultural and ethical dimensions of patient care.

*“Negative capability helped me see that sitting with uncertainty is not weakness but an important skill.” (Student 2023)*

#### Cross-mechanism patterns: A sequential learning pathway

Across both cohorts, mechanisms did not operate in isolation. Instead, the following sequential pattern emerged that represents a developing middle-range theory explaining how behavioral science education shapes students’ cognitive and emotional engagement:

1. **Emotional articulation** enabled
2. **Narrative destabilization**, which created space for
3. **Positional reframing**, leading to
4. **Epistemic disruption**, and ultimately
5. **Conceptual reframing and meaning-making**.

This pattern refines our iPTs by demonstrating that mechanisms were activated progressively rather than independently.

## Discussion

This realist evaluation identified five learning mechanisms that explain how a behavioral science course supported medical students’ cognitive, emotional, and relational development. These mechanisms—emotional articulation, narrative destabilization, positional reframing, epistemic disruption, and conceptual reframing—operate not as isolated components of an intervention but as internal processes triggered under specific contextual conditions. Our findings refine the iPTs and offer a middle-range theory describing how students learn to engage more reflectively and empathetically with the complexities of clinical practice [22].

### Mechanisms as sequential and interdependent processes

A key contribution of this study is the identification of a sequential learning pathway. Emotional articulation served as an essential starting point, enabling students to participate in discussions with greater openness and reduced defensiveness [3,23,24]. This foundation allowed narrative destabilization to unsettle biomedical certainties, creating the cognitive space needed for positional reframing [10]. In turn, positional reframing made epistemic disruption tolerable, rather than overwhelming [25]. Finally, conceptual reframing allowed students to re-interpret their discomfort and uncertainty as meaningful components of professional identity formation [17,26].

This chain of mechanisms demonstrates that learning in behavioral science education unfolds cumulatively and relationally, rather than through independent, modular components. Notably, this sequential pattern helps explain why educational activities such as case discussions, when delivered in isolation, often fail to generate deep reflection. Without adequate emotional scaffolding, learners tend to adopt defensive stances that limit the activation of reflective mechanisms [23,27]. Prior emotional articulation thus creates the psychological readiness required for narrative encounters to become genuinely transformative [28].

### Slow Education as a sensitizing concept

**Figure 1:**
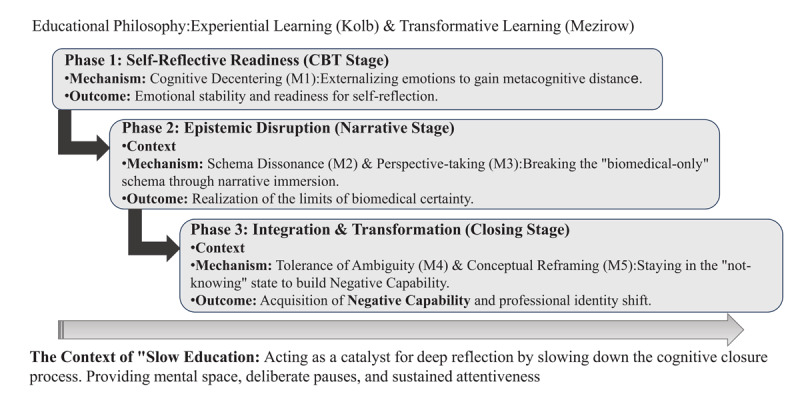
The Sequential Scaffolding of Behavioral Science Education: A Realist Evaluation Model.

In our evaluation, ‘Time’ emerged not merely as a duration but as a critical contextual enabler. Within the high-pressure, “fast-paced” context of medical school, the deliberate “slowness” of our curriculum—characterized by strategic pauses and reflection-in-action—functioned as a catalyst for Mechanism 4 (Tolerance of Ambiguity). By slowing down the cognitive process, students were afforded the “mental space” required to resist premature closure and develop the negative capability necessary for complex clinical reasoning. As illustrated in Figure 1, this “slowness” serves as an overarching contextual foundation that facilitates the sequential transition between mechanisms, ensuring that students have the psychological capacity to move from initial reflection to deep epistemic transformation. Slow Education functioned effectively as a sensitizing concept that guided both course design and theory development [13,29]. Its emphasis on intentional pausing, narrative dwelling, and engaging with uncertainty helped clarify why the learning mechanisms were activated only when students were given sufficient time and psychological space to process their experiences. This finding aligns with work showing that deep reflection requires temporal and emotional openness [27,30], that dwelling on the narrative expands interpretive possibilities [31], and that uncertainty can be productively engaged only when psychological safety is present [32,33]. The visual representation of this process in Figure 1 demonstrates that the activation of reflective mechanisms is not immediate but depends on a cumulative scaffolding process sustained by deliberate temporal pacing. The findings suggest that “slowing down”—through structured reflection, prolonged engagement with narratives, and timely conceptual input—is not merely a philosophical aspiration but a practical design principle for triggering mechanisms of reflective learning.

### Competing and unactivated mechanisms

Realist evaluation requires explaining not only what works but also why intended mechanisms may fail to activate [14,34]. In our study, a competing certainty-seeking mechanism emerged in a subset of students who reverted to biomedical correctness or withdrew from ambiguous discussion. Prior mechanisms—such as emotional articulation or narrative destabilization—remained unactivated, consistent with research showing that rigid epistemic norms, performance anxiety, and limited reflective experience can suppress learners’ capacity to engage with uncertainty [27,33,35]. Recognizing these non-activations is critical for refining educational design. Students who experience epistemic disruption as distress rather than generative discomfort may require additional scaffolding to support transformative engagement [23,36].

### Theoretical implications

This study contributes to the broader literature on behavioral science and health professions education by clarifying how reflective capacity, perspective-taking, and tolerance of ambiguity develop in early-stage medical trainees. Specifically:

- Emotional articulation supports reflective practice theories by demonstrating the importance of making implicit emotional reactions explicit.
- Narrative destabilization aligns with theories of narrative identity formation and highlights the role of lived experience in reshaping clinical reasoning.
- Positional reframing extends sociocultural learning theories, showing how structured dialogue facilitates shifts in interpretive stance.
- Epistemic disruption resonates with threshold concepts and troublesome knowledge, indicating moments when learners confront the boundaries of biomedical epistemology.
- Conceptual reframing demonstrates how theoretical tools support meaning-making, helping students re-narrate their professional identities.

Collectively, these mechanisms advance a middle-range theory of how behavioral science education can cultivate the reflective, relational, and uncertainty-tolerant capacities needed in contemporary clinical practice.

### Practical implications for curriculum design

The findings suggest several transferable design principles for educators:

1. **Begin with emotional scaffolding**
  Structured reflection helps students engage more openly with complex narratives.
2. **Use rich, ambiguous cases rather than reductive vignettes**
  Narrative complexity is a catalyst for destabilizing narrow biomedical assumptions.
3. **Integrate structured questioning**
  Questions that challenge assumptions and prompt imaginative perspective-taking deepen interpretive work.
4. **Include deliberately unresolved cases**
  Ambiguity is essential for activating epistemic disruption and developing tolerance for uncertainty.
5. **Provide conceptual tools at moments of heightened reflection**
  Timely introduction of sociocultural or humanistic concepts gives students language to make sense of their experiences.

These strategies are culturally adaptable and can be applied across diverse medical education settings.

## Limitations

This study is limited by its single-institution context and reliance on self-reported written reflections. Students’ developmental readiness for reflective work likely varied, influencing the activation of certain mechanisms. Future research should examine how these mechanisms operate longitudinally and across different cultural or institutional environments, including whether the sequential learning pathway generalizes to other educational contexts.

## Conclusion

By identifying how and under what conditions mechanisms of emotional articulation, narrative destabilization, positional reframing, epistemic disruption, and conceptual reframing are activated, this study provides a robust explanatory account of learning within behavioral science education. The resulting middle-range theory offers a practical and transferable framework for designing curricula that foster reflection, empathy, and tolerance for ambiguity—competencies essential for navigating the complexities of modern clinical practice.

## Additional File

The additional file for this article can be found as follows:

10.5334/pme.2332.s1 Additional file 1. Illustrative student excerpts supporting each CMO configuration.

## Data Availability

The dataset analyzed during the current study are available from the corresponding author on reasonable request.
