# Supplementary material for: How Time, Narrative, and Discomfort Shape Learning: A Realist Evaluation of Behavioral Science Education for Medical Students
Source: Perspect Med Educ. 2026 May 5;15(1):397–409. doi: 10.5334/pme.2332 (PMC13155095; doi:10.5334/pme.2332)
Supplement: Additional file 1. — Illustrative student excerpts supporting each CMO configuration. [file pme-15-1-2332-s1.pdf]

Additional file 1: Illustrative student excerpts supporting each CMO configuration

Mechanism No.	Representative Data (Excerpted):
1	*Student A（2023）: I learned that the cognitive behavioral model is built on the idea that cognition affects mood and behavior. Writing about a time I felt frustrated during club activities helped me see how my assumptions shaped my reactions.*<br>*Student B （2024）: By identifying the thoughts behind my irritation toward a peer, I realized I could reframe the situation and respond more constructively to patients. As a future physician, I realized the importance of being mindful of my own mental health. Using CBT helped me reflect and manage negative thought patterns.*
2	*Student C (2023): Reading the case of a lawyer with Guillain-Barré syndrome who later faced stomach cancer made me realize how differently patients may perceive treatments. Through group discussion, I recognized the importance of understanding patient backgrounds and perspectives.*

	Student D (2024): I learned the value of imagining patients' lived realities. Simply seeing "non-adherence" to medication might evoke frustration, but imagining the reasons behind such behaviors fosters empathy and better care.
3	*Student E (2023): What I learned most in behavioral science was how, by studying in a closed environment like medical school and later working in a highly specialized healthcare setting, I may unconsciously lose the "patient's perspective." To remain a doctor who can see patients in a balanced way, I realized the importance of having regular opportunities—like this course—to deeply consider patients' backgrounds. I spend much of my time in the lab doing research, immersed in highly specialized discussions even more than typical clinical settings, which makes me even more prone to losing sight of general perspectives. If we are to advance medicine and save more patients, we must intentionally make time to broaden our view.*<br>*Student F (2024): I learned that when I'm truly stuck as a physician, it's essential to involve more people in the*

	problem-solving process. Also, neither patients nor their caregiving families should be left isolated. I came to understand that even the best treatments are less effective when someone is socially isolated.<br>*Student G (2024): We are repeatedly taught from elementary school that it's important to view things from various perspectives, and I've always believed that. However, through this behavioral science course, I realized I wasn't truly seeing things from multiple angles. I tended to interpret things through the lens of my own values and beliefs, and I noticed that I had unconsciously excluded perspectives I couldn't understand or imagine.*
4	*Student H (2023): In other courses, we primarily focus on diagnostics and treatment protocols, so I've come to believe that the most important thing is to provide evidence-based, scientifically appropriate care. But in behavioral science, I came to recognize how critical the emotional bond between doctor and patient is to successful treatment outcomes. I want to explore how best to build trust in daily relationships. I was particularly struck by the realization that patients may hold values completely different from any I've encountered. As a*

	physician, even when facing value differences with patients, we must rationally understand their values and decide on a treatment plan, but I can easily imagine myself reacting emotionally. I want to keep in mind that the values I know are only a small part of the spectrum.<br>*Student I (2024): I felt how difficult it is to provide truly patient-centered care, especially in end-of-life settings. Balancing a patient's wishes against what is medically right is a tough issue. Respecting a patient's autonomy and how they want to live their remaining life can offer peace and dignity. But if the patient's choices aren't medically appropriate, it could lead to deterioration and more suffering. If their decisions also burden their family, relationships might suffer. On the other hand, focusing on medically "correct" care helps improve the physical condition and makes treatment easier from the physician's standpoint. However, that could lead to ignoring the patient's wishes, eroding trust. To resolve this, I think flexibility is necessary—listening closely to the patient's reasoning and negotiating the best path forward. In the case we studied, the physician didn't force*

	treatment but respected the patient's stance, which helped build trust and led to satisfactory care. I realized that in end-of-life care, what's medically right isn't always the top priority for the patient.
5	Student J (2023): I felt I understood the meaning of the term "troublesome knowledge" mentioned by our instructor. In the case presented, the focus was not on the disease itself but on the patient's family dynamics and social background. It would be ideal to understand those aspects easily, but in real home visits, it takes time and persistent dialogue to earn trust and grasp such complexity. I recalled a passage from the book "Being Here is Painful," which said that what's most important in connecting hearts isn't diagnosing or treating a disease but simply being present and becoming part of that space. To truly understand what matters, it's not about efficiency—it requires time and patience.<br><br>Student L (2024): The behavioral science assignments didn't have clear answers, so I had to think deeply from

	various angles, which was often challenging. I learned about the term “negative capability” for the first time and felt it would be a necessary trait for me as a future physician. We had many opportunities for discussion, and I realized how diverse perspectives can be, even among classmates. I want to become someone who, when faced with unsolvable issues, values dialogue with fellow doctors, interprofessional colleagues, patients, and families, and can consider problems from many dimensions
